# The ribosomal protein genes and *Minute *loci of *Drosophila melanogaster*

**DOI:** 10.1186/gb-2007-8-10-r216

**Published:** 2007-10-10

**Authors:** Steven J Marygold, John Roote, Gunter Reuter, Andrew Lambertsson, Michael Ashburner, Gillian H Millburn, Paul M Harrison, Zhan Yu, Naoya Kenmochi, Thomas C Kaufman, Sally J Leevers, Kevin R Cook

**Affiliations:** 1 Growth Regulation Laboratory, Cancer Research UK London Research Institute, Lincoln's Inn Fields, London WC2A 3PX, UK; 2 Department of Genetics, University of Cambridge, Downing Street, Cambridge CB2 3EH, UK; 3 Institute of Genetics, Biologicum, Martin Luther University Halle-Wittenberg, Weinbergweg, Halle D-06108, Germany; 4 Institute of Molecular Biosciences, University of Oslo, Blindern, Olso N-0316, Norway; 5 Department of Biology, McGill University, Dr Penfield Ave, Montreal, Quebec H3A 1B1, Canada; 6 Frontier Science Research Center, University of Miyazaki, 5200 Kihara, Kiyotake, Miyazaki 889-1692, Japan; 7 Department of Biology, Indiana University, E. Third Street, Bloomington, IN 47405-7005, USA

## Abstract

**Background:**

Mutations in genes encoding ribosomal proteins (RPs) have been shown to cause an array of cellular and developmental defects in a variety of organisms. In *Drosophila melanogaster*, disruption of RP genes can result in the 'Minute' syndrome of dominant, haploinsufficient phenotypes, which include prolonged development, short and thin bristles, and poor fertility and viability. While more than 50 *Minute *loci have been defined genetically, only 15 have so far been characterized molecularly and shown to correspond to RP genes.

**Results:**

We combined bioinformatic and genetic approaches to conduct a systematic analysis of the relationship between RP genes and *Minute *loci. First, we identified 88 genes encoding 79 different cytoplasmic RPs (CRPs) and 75 genes encoding distinct mitochondrial RPs (MRPs). Interestingly, nine CRP genes are present as duplicates and, while all appear to be functional, one member of each gene pair has relatively limited expression. Next, we defined 65 discrete *Minute *loci by genetic criteria. Of these, 64 correspond to, or very likely correspond to, CRP genes; the single non-CRP-encoding *Minute *gene encodes a translation initiation factor subunit. Significantly, MRP genes and more than 20 CRP genes do not correspond to *Minute *loci.

**Conclusion:**

This work answers a longstanding question about the molecular nature of *Minute *loci and suggests that Minute phenotypes arise from suboptimal protein synthesis resulting from reduced levels of cytoribosomes. Furthermore, by identifying the majority of haplolethal and haplosterile loci at the molecular level, our data will directly benefit efforts to attain complete deletion coverage of the *D. melanogaster *genome.

## Abstract 2

A combined bioinformatic and genetic approach was used to conduct a systematic analysis of the relationship between ribosomal protein genes and Minute loci in Drosophila melanogaster, allowing the identification of 64 Minute loci corresponding to ribosomal genes.

## Background

Ribosomes are sophisticated macromolecular machines that catalyze cellular protein synthesis in all cells of all organisms. They have an ancient evolutionary origin and are essential for cell growth, proliferation and viability. Though larger and more complex in higher organisms, both the structure and function of ribosomes have been conserved throughout evolution. Genetic approaches in *Drosophila melanogaster *have shown that disrupting ribosome function can result in an array of fascinating dominant phenotypes [1,2]. Despite this, there has so far been no comprehensive inventory of genes encoding ribosome components in this organism, nor any systematic effort to determine their mutant phenotypes.

All ribosomes comprise a set of ribosomal proteins (RPs) surrounding a catalytic core of ribosomal RNA (rRNA). Bacteria possess a single type of ribosome composed of three rRNA molecules and typically 54 RPs. All eukaryotic cells, in contrast, contain at least two distinct types of ribosomes: cytoplasmic ribosomes (cytoribosomes) and mitochondrial ribosomes (mitoribosomes). Cytoribosomes are found on the endoplasmic reticulum and in the aqueous cytoplasm. They translate all mRNAs produced from nuclear genes and perform the vast majority of cellular protein synthesis. Each cytoribosome contains four different rRNAs and 78-80 cytoplasmic RPs (CRPs). Mitoribosomes consist of only two rRNA molecules and up to 80 mitochondrial RPs (MRPs). They are located in the mitochondrial matrix and synthesize proteins involved in oxidative phosphorylation encoded by those few genes retained in the mitochondrial genome. A third unique type of eukaryotic ribosome is found within the plastids (for example, chloroplasts) of plant and various algal cells. In all cases, distinct small and large ribosomal subunits exist that join together during the translation initiation process to form mature ribosomes capable of protein synthesis. (See references [3-6] for general reviews of ribosomal structure and function.)

The protein components of ribosomes are interesting from several points of view. First, and most obviously, RPs play critical roles in ribosome assembly and function [7]. Second, several RPs perform important extra-ribosomal functions, including roles in DNA repair, transcriptional regulation and apoptosis [6,8]. Third, misexpression of human CRP and MRP genes has been implicated in a wide spectrum of human syndromes and diseases, including Diamond-Blackfan anaemia [9], Turner syndrome [10], hearing loss [11] and cancer [12]. Fourth, mutations in the CRP genes of *D. melanogaster *are important tools for the study of growth, development and cell competition [2]. Finally, many RPs are conserved from bacteria to humans, so their peptide and nucleotide sequences are useful for studying phylogenetic relationships [13].

The first eukaryotic CRPs characterized in detail were isolated from the rat cytoribosome [3]. Individual proteins were separated by two-dimensional gel electrophoresis and named from their origin in the small (S) or large (L) subunit and their relative electrophoretic migration positions, for example, RPS9 or RPL28. Subsequent studies revealed that some protein spots contained non-ribosomal proteins or chemically modified versions of another CRP, and that some spots contained two co-migrating CRPs [3,5]. Consequently, the nomenclature system used today contains numerical gaps as well as 'A' suffixes for those additional CRPs not resolved by the original electrophoresis (for example, RPL36A). Seventy-nine distinct mammalian CRPs are now acknowledged and their amino acid sequences and biochemical properties have been described [5,14]. With the exception of RPLP1 and RPLP2, each of which forms homodimers in the cytoribosomal large subunit [15], all CRPs are present as single molecules in each cytoribosome [3].

Seventy-eight different mammalian MRPs have been described [6] and their individual amino acid sequences and biochemical properties have been determined [16,17]. Although the nomenclature of MRPs was originally based on electrophoretic properties, the current system reflects homology between mammalian MRPs and their bacterial orthologs [18]. Thus, MRPS1 through MRPS21 are orthologous to *Escherichia coli *RPs S1-S21, while higher numbers have been assigned to the MRPs not found in bacteria. Gaps also exist in MRP numbering because a gap occurs in the bacterial enumeration or because there is no mammalian ortholog.

The RPs of *D. melanogaster *were first studied in the 1970s and early 1980s. Up to 78 individual CRPs were observed on two-dimensional gels [19-31] and about 30 were purified and analyzed biochemically [32,33]. A more recent characterization used mass spectrometry to identify 52 *D. melanogaster *CRPs [34], all of which are orthologous to known mammalian CRPs. The protein composition of *Drosophila *mitoribosomes has not been characterized biochemically to date.

CRPs and MRPs are encoded by the nuclear genome. Knowledge of the primary sequences of rat CRPs and bovine MRPs has led to the identification and mapping of the RP-encoding genes in many eukaryotic species [14]. Indeed, systematic analyses of whole RP gene sets have been described for several organisms, including *Saccharomyces cerevisiae *[35], *Arabidopsis thaliana *[36] and humans [37-40]. However, the complete set of *D. melanogaster *CRP and MRP genes has not been previously documented or characterized.

Several *D. melanogaster *RP genes were initially identified by virtue of their dominant 'Minute' mutant phenotypes [2], which include prolonged development, low fertility and viability, altered body size and abnormally short, thin bristles on the adult body. All of these phenotypes may be explained by a cell-autonomous defect in protein biosynthesis: the production of each bristle, for example, requires a very high rate of protein synthesis in a single cell during a short developmental period. Merriam and colleagues reported the first unequivocal molecular link between a *Minute *locus and a CRP gene in 1985 [41]. Since then, 14 additional *Minute *loci have been definitively linked with distinct CRP genes [2,42-53]. However, there are at least 35 genetically validated *Minute *loci that have not yet been associated with a specific gene and there may be additional *Minute *genes to be discovered. Several investigators have hypothesized that all *Minute *loci encode protein components of ribosomes (reviewed in [2]). Whether this is truly the case and whether both CRP and MRP genes are associated with Minute phenotypes are open and intriguing questions.

Many *Minute *loci were originally identified from the phenotypes of flies heterozygous for a chromosomal deletion [54,55] and all *Minute *point mutations studied in depth have been found to be loss-of-function alleles [2]. This indicates that Minute phenotypes can be attributed to genetic haploinsufficiency; that is, a single gene copy is not sufficient for normal development. (Note that *X*-linked mutations that cause Minute phenotypes in heterozygous females are lethal in hemizygous males.) The most popular explanation for the haploinsufficiency of *Minute *loci is that they correspond to RP genes and that RPs are required in equimolar amounts: halving the copy number of a single RP gene limits the availability of the encoded RP, thereby reducing the number of functional ribosomes that are assembled in the cell and impairing protein synthesis [2]. While this idea is consistent with the available data, there may be other explanations.

The reduced fertility and viability associated with many *Minute *loci makes the recovery of deletions uncovering them rather difficult - the mutant strains are too weak to maintain as stable heterozygous stocks. In fact, some *Minute *loci are known only from the phenotypes of transient aneuploids [54,56]. This means that several chromosomal regions containing a *Minute *locus are not uncovered by current deletion collections [57]. This is frustrating for researchers because deletions are basic tools for mutational analysis and are widely used for mapping new mutations and identifying genetic modifiers. Efforts to maximize deletion coverage of the *D. melanogaster *genome would benefit from a systematic assessment of the relationship between RP genes and *Minute *loci. It would allow the isolation of deletions that flank haploinsufficient RP genes as closely as possible, or the design of transgenic constructs or chromosomal duplications to rescue the haploinsufficiency of deletions uncovering *Minute *genes.

Here, we report the systematic identification, naming and characterization of all the CRP and MRP genes of *D. melanogaster*. We have used this information, together with phenotypic data obtained from examining mutation and deficiency strains, to assess the correspondence between RP genes and *Minute *loci. We find that 66 of the 88 CRP genes identified are, or are very likely to be, haploinsufficient and associated with a Minute phenotype, whereas MRP genes and the remaining 22 CRP genes are not. Significantly, we show that all but one of the known *Minute *loci in the genome correspond to CRP genes - the single exception encodes a subunit of an essential translation initiation factor. Together, these results identify the majority of haploinsufficient loci in the *D. melanogaster *genome that significantly affect viability, fertility and/or external morphology, and also provide a mechanistic framework for understanding the Minute syndrome and the phenotypic effects of aneuploidy.

## Results

### Identification of *D. melanogaster *ribosomal protein genes

In order to conduct an exhaustive survey of *Drosophila *CRP and MRP genes, we first performed a series of BLAST searches using human RP sequences as queries, because both CRPs and MRPs have been well-characterized in humans [5,6]. Tables 1 and 2 list the genes we identified together with their cytological locations. Where necessary, *D. melanogaster *genes were named or renamed according to the standard metazoan RP gene nomenclature proposed by Wool and colleagues [5,58,59] and approved by the HUGO Gene Nomenclature Committee [18], whilst still conforming to FlyBase [60] conventions - that is, CRP genes are given an '*Rp*' prefix and MRP genes have an '*mRp*' prefix. The seven exceptions to this standard RP nomenclature are mostly genes originally named to reflect a mutant phenotype, for example, the *string of pearls *(*sop*) gene encodes RpS2 [61] and *bonsai *encodes mRpS15 [62,63]. In these cases, the original gene symbol has been preserved, with the apposite RP symbol given as a synonym.

**Table 1:** The CRP genes of *D. melanogaster*

	*D. melanogaster *gene			
		
Human CRP	Symbol*	CG number	Location^†^	BLAST E value^‡^
RPSA	*sta/RpSA*	*CG14792*	X: 2B1	3e-94
RPS2	*sop/RpS2*	*CG5920*	2L: 30E1	e-118
RPS3	*RpS3*	*CG6779*	3R: 94E13	e-106
RPS3A	*RpS3A*	*CG2168*	4: 101F1	e-100
RPS4	*RpS4*	*CG11276*	3L: 69F6	e-121
RPS5	*RpS5a*	*CG8922*	X: 15E5-7	1e-98
	***RpS5b***	*CG7014*	3R: 88D6	3e-96
RPS6	*RpS6*	*CG10944*	X: 7C2	e-103
	***CG11386***	*CG11386*	X: 7C2	2e-15
	***CG33222***	*CG33222*	X: 7C2	2e-15
RPS7	*RpS7*	*CG1883*	3R: 99E2	1e-74
RPS8	*RpS8*	*CG7808*	3R: 99C4	1e-82
RPS9	*RpS9*	*CG3395*	3L: 67B11	8e-92
RPS10	***RpS10a***	*CG12275*	3R: 98A14	4e-43
	*RpS10b*	*CG14206*	X: 18D3	8e-52
RPS11	*RpS11*	*CG8857*	2R: 48E8-9	1e-58
RPS12	*RpS12*	*CG11271*	3L: 69F5	2e-43
RPS13	*RpS13*	*CG13389*	2L: 29B2	2e-74
RPS14	*RpS14a*	*CG1524*	X: 7C6-7	3e-71
	***RpS14b***	*CG1527*	X: 7C8	3e-71
RPS15	*RpS15*	*CG8332*	2R: 53C8	6e-62
RPS15A	*RpS15Aa*	*CG2033*	X: 11E11-12	2e-65
	***RpS15Ab***	*CG12324*	2R: 47C1	6e-65
RPS16	*RpS16*	*CG4046*	2R: 58F1	2e-69
RPS17	*RpS17*	*CG3922*	3L: 67B5	5e-52
RPS18	*RpS18*	*CG8900*	2R: 56F11	2e-69
RPS19	*RpS19a*	*CG4464*	X: 14F4	5e-48
	***RpS19b***	*CG5338*	3R: 95C13	1e-43
RPS20	*RpS20*	*CG15693*	3R: 93A1	7e-50
RPS21	*oho23B/RpS21*	*CG2986*	2L: 23B6	3e-30
RPS23	*RpS23*	*CG8415*	2R: 50E4	8e-70
RPS24	*RpS24*	*CG3751*	2R: 58F3	7e-55
RPS25	*RpS25*	*CG6684*	3R: 86D8	1e-38
RPS26	*RpS26*	*CG10305*	2L: 36F4	3e-47
RPS27	*RpS27*	*CG10423*	3R: 96C8	2e-39
RPS27A	*RpS27A*	*CG5271*	2L: 31E1	8e-80
RPS28	***RpS28a***	*CG15527*	3R: 99D2	1e-21
	*RpS28b*	*CG2998*	X: 8E7	2e-23
	***RpS28-like***	*CG34182*	2L: 30B3	1e-07
RPS29	*RpS29*	*CG8495*	3R: 85E8	5e-23
RPS30	*RpS30*	*CG15697*	3R: 93A2	6e-32
				
RPLP0	*RpLP0*	*CG7490*	3L: 79B2	e-122
	***RpLP0-like***	*CG1381*	2R: 46E5-6	3e-10
RPLP1	*RpLP1*	*CG4087*	2L: 21C2	3e-34
RPLP2	*RpLP2*	*CG4918*	2R: 53C9	5e-32
RPL3	*RpL3*	*CG4863*	3R: 86D8	0.0
RPL4	*RpL4*	*CG5502*	3R: 98B6	e-141
RPL5	*RpL5*	*CG17489*	2L: h35/40B	e-120
RPL6	*RpL6*	*CG11522*	3R: 100C7	8e-58
RPL7	*RpL7*	*CG4897*	2L: 31B1	2e-75
	***RpL7-like***	*CG5317*	2L: 33C1	3e-32
RPL7A	*RpL7A*	*CG3314*	X: 6B1	e-102
RPL8	*RpL8*	*CG1263*	3L: 62E7	e-119
RPL9	*RpL9*	*CG6141*	2L: 32C1	1e-66
RPL10	*Qm/RpL10*	*CG17521*	3L: h47/80A	7e-98
RPL10A	***RpL10Aa***	*CG3843*	3R: 88D10	3e-65
	*RpL10Ab*	*CG7283*	3L: 68E1	3e-95
RPL11	*RpL11*	*CG7726*	2R: 56D7	6e-83
RPL12	*RpL12*	*CG3195*	2R: 60B7	7e-75
RPL13	*RpL13*	*CG4651*	2L: 30F3	1e-68
RPL13A	*RpL13A*	*CG1475*	3R: 83B6-7	3e-68
RPL14	*RpL14*	*CG6253*	3L: 66D8	2e-30
RPL15	*RpL15*	*CG17420*	3L: h50-52/80F	5e-90
RPL17	*RpL17*	*CG3203*	X: 6C10	5e-72
RPL18	*RpL18*	*CG8615*	3L: 65E9	3e-71
RPL18A	*RpL18A*	*CG6510*	2R: 54C3	3e-68
RPL19	*RpL19*	*CG2746*	2R: 60E11	4e-83
RPL21	*RpL21*	*CG12775*	2L: 40A-B	4e-66
RPL22	*RpL22*	*CG7434*	X: 1C4	4e-40
	***RpL22-like***	*CG9871*	2R: 59D3	2e-24
RPL23	*RpL23*	*CG3661*	2R: 59B3	1e-68
RPL23A	*RpL23A*	*CG7977*	3L: 62A10	7e-52
RPL24	*RpL24*	*CG9282*	2L: 34B10	7e-55
	***RpL24-like***	*CG6764*	3R: 86E5	8e-14
RPL26	*RpL26*	*CG6846*	3L: 75E4	1e-59
RPL27	*RpL27*	*CG4759*	3R: 96E9-10	1e-43
RPL27A	*RpL27A*	*CG15442*	2L: 24F3	5e-60
RPL28	*RpL28*	*CG12740*	3L: 63B14	3e-31
RPL29	*RpL29*	*CG10071*	2R: 57D8	8e-14
RPL30	*RpL30*	*CG10652*	2L: 37B9	3e-46
RPL31	*RpL31*	*CG1821*	2R: 45F5	5e-47
RPL32	*RpL32*	*CG7939*	3R: 99D3	6e-58
RPL34	***RpL34a***	*CG6090*	3R: 96F10	2e-29
	*RpL34b*	*CG9354*	3R: 85D15	1e-29
RPL35	*RpL35*	*CG4111*	X: 5A11	2e-38
RPL35A	*RpL35A*	*CG2099*	3R: 83A4	1e-35
RPL36	*RpL36*	*CG7622*	X: 1B12	3e-33
RPL36A	*RpL36A*	*CG7424*	2L: 28D3	3e-43
RPL37	*RpL37a*	*CG9091*	X: 13B1	2e-39
	***RpL37b***	*CG9873*	2R: 59C4	1e-31
RPL37A	*RpL37A*	*CG5827*	2L: 25C4	2e-38
RPL38	*RpL38*	*CG18001*	2R: h46/41C-E	2e-25
RPL39	*RpL39*	*CG3997*	2R: 60B7	3e-18
RPL40	*RpL40*	*CG2960*	2L: 24E1	2e-69
RPL41	*RpL41*	*CG30425*	2R: 60E5	8e-08

*Additional gene synonyms exist in most cases [60]. Bold font indicates CRP-like genes, putative pseudogenic fragments (*CG11386 *and *CG33222*) or the member of a duplicate gene pair that is expressed in a small number of tissues and/or at relatively low levels. ^†^Computed cytological position is given for euchromatic genes (Genome Release 5 [60]). The cytological and h-band locations for heterochromatic genes are based on data in reference [151] or estimated from images of *in situ *hybridizations of BACs to polytene chromosomes (*RpL5 *and *RpL21*) [152]. The h-band location of *RpL15 *was provided by B Honda (personal communication). ^‡^Expect (E) value obtained from a BLASTp search of the *D. melanogaster *annotated proteome (Genome Release 5.1) with human RefSeq CRP sequences. (E values corresponding to *RpL15 *and *RpS28-like *were obtained from a BLAST search using Release 5.3.) Where multiple protein isoforms exist, the highest scoring hit is given.

**Table 2:** The MRP genes of *D. melanogaster*

	*D. melanogaster *gene			
		
Human MRP	Symbol*	CG number	Location^†^	BLAST E value^‡^
MRPS2	*mRpS2*	*CG2937*	2L: 25B1	1e-69
MRPS5	*mRpS5*	*CG40049*	3L: h47/80A-B	4e-63
MRPS6	*mRpS6*	*CG15016*	3L: 64B2	2e-20
MRPS7	*mRpS7*	*CG5108*	2L: 31D11	6e-38
MRPS9	*mRpS9*	*CG2957*	3R: 84E4	8e-81
MRPS10	*mRpS10*	*CG4247*	3R: 88E3	1e-33
MRPS11	*mRpS11*	*CG5184*	3R: 89E11	8e-26
MRPS12	*tko/mRpS12*	*CG7925*	X: 3A3	1e-33
MRPS14	*mRpS14*	*CG32531*	X: 18C7	1e-35
MRPS15	*bonsai/mRpS15*	*CG4207*	2R: 58F3	1e-15
MRPS16	*mRpS16*	*CG8338*	2R: 50E1	2e-24
MRPS17	*mRpS17*	*CG4326*	2R: 60C1	2e-14
MRPS18A	*mRpS18A*	*CG31450*	3R: 85A3	8e-13
MRPS18B	*mRpS18B*	*CG10757*	2L: 38B6	4e-33
MRPS18C	*mRpS18C*	*CG9688*	3R: 99F4	7e-23
MRPS21	*mRpS21*	*CG32854*	3R: 87E8	3e-22
MRPS22	*mRpS22*	*CG12261*	3R: 98B6	3e-38
MRPS23	*mRpS23*	*CG31842*	2L: 34D6	2e-20
MRPS24	*mRpS24*	*CG13608*	3R: 95E6	2e-31
MRPS25	*mRpS25*	*CG14413*	X: 12F1	1e-49
MRPS26	*mRpS26*	*CG7354*	3L: 75B9	3e-11
MRPS27	NA	NA	NA	No hit
MRPS28	*mRpS28*	*CG5497*	2R: 55E2	1e-27
DAP3/MRPS29	*mRpS29*	*CG3633*	2R: 58E1	7e-72
MRPS30	*mRpS30*	*CG8470*	X: 13E18	3e-21
MRPS31	*mRpS31*	*CG5904*	3L: 72C2	5e-35
MRPS33	*mRpS33*	*CG10406*	3R: 89B16	3e-30
MRPS34	*mRpS34*	*CG13037*	3L: 72E1-2	8e-06
MRPS35	*mRpS35*	*CG2101*	3L: 62F4	2e-72
MRPS36	NA	NA	NA	No hit
				
MRPL1	*mRpL1*	*CG7494*	3R: 84F9-10	8e-25
MRPL2	*mRpL2*	*CG7636*	3L: 68A7	2e-56
MRPL3	*mRpL3*	*CG8288*	X: 13E14	5e-52
MRPL4	*mRpL4*	*CG5818*	2L: 35F1	6e-70
MRPL9	*mRpL9*	*CG31478*	3R: 88F1	6e-17
MRPL10	*mRpL10*	*CG11488*	2L: 21B4	8e-20
MRPL11	*mRpL11*	*CG3351*	3R: 88C3	4e-29
MRPL12	*mRpL12*	*CG5012*	3L: 66E5	2e-13
MRPL13	*mRpL13*	*CG10603*	2L: 37B1	2e-47
MRPL14	*mRpL14*	*CG14048*	X: 3A1	3e-31
MRPL15	*mRpL15*	*CG5219*	3L: 77C3	4e-74
MRPL16	*mRpL16*	*CG3109*	X: 2B14	3e-58
MRPL17	*mRpL17*	*CG13880*	3L: 61B3	8e-21
MRPL18	*mRpL18*	*CG12373*	2R: 49C2	3e-22
MRPL19	*mRpL19*	*CG8039*	3R: 85A5	1e-56
MRPL20	*mRpL20*	*CG11258*	3L: 69F5	8e-21
MRPL21	*mRpL21*	*CG9730*	3L: 76A3	2e-19
MRPL22	*mRpL22*	*CG4742*	X: 15A7-8	4e-41
MRPL23	*mRpL23*	*CG1320*	3L: 62D7	4e-28
MRPL24	*mRpL24*	*CG8849*	2L: 25B4	2e-45
MRPL27	*mRpL27*	*CG33002*	2L: 24F3	2e-10
MRPL28	*mRpL28*	*CG3782*	2L: 25B5	5e-27
MRPL30	*mRpL30*	*CG7038*	X: 4C11	3e-15
MRPL32	*mRpL32*	*CG12220*	3R: 100B8	6e-15
MRPL33	*mRpL33*	*CG3712*	X: 4B6	5e-08
MRPL34	*mRpL34*	*CG34147*	2R: 52E4	1e-07
MRPL35	*mRpL35*	*CG13410*	3R: 94A1	4e-25
MRPL36	*mRpL36*	*CG18767*	3L: 66B7	2e-09
MRPL37	*mRpL37*	*CG6547*	3R: 86C6	4e-16
MRPL38	*mRpL38*	*CG15871*	X: 12E5	2e-61
MRPL39	*mRpL39*	*CG17166*	3L: 71B1	2e-57
MRPL40	*mRpL40*	*CG5242*	3R: 86E4	6e-13
MRPL41	*mRpL41*	*CG12954*	2R: 51E7	2e-11
MRPL42	*mRpL42*	*CG12921*	2R: 46E1	1e-11
MRPL43	*mRpL43*	*CG5479*	2R: 59F6	1e-25
MRPL44	*mRpL44*	*CG2109*	3R: 83A4	6e-39
MRPL45	*mRpL45*	*CG6949*	3R: 94B6	4e-61
MRPL46	*mRpL46*	*CG13922*	3L: 62B4	5e-39
MRPL47	*Rlc1/mRpL47*	*CG9378*	3R: 85D19	1e-35
MRPL48	*mRpL48*	*CG17642*	2L: 22B1	4e-15
MRPL49	*mRpL49*	*CG4647*	X: 11D1	9e-22
MRPL50	*mRpL50*	*CG8612*	3L: 65E9	6e-09
MRPL51	*mRpL51*	*CG13098*	2L: 29D4	2e-13
MRPL52	*mRpL52*	*CG1577*	2R: 43E9	2e-14
MRPL53	*mRpL53*	*CG30481*	2R: 50C16	3e-06
MRPL54	*mRpL54*	*CG9353*	2R: 57B16	2e-18
MRPL55	*mRpL55*	*CG14283*	3R: 91F1	3e-17
LACTB/MRPL56	NA	NA	NA	No hit

*Additional gene synonyms exist in many cases [60]. ^†^Computed cytological position is given for euchromatic genes (Genome Release 5 [60]). *mRpS5 *h-band location provided by C Smith (DHGP, personal communication) and cytological position inferred from reference [151]. ^‡^Expect (E) value obtained from a BLASTp search of the *D. melanogaster *annotated proteome (Genome Release 5.1) with human RefSeq MRP sequences. Where multiple protein isoforms exist, the highest scoring hit is given.

### Cytoplasmic ribosomal protein genes

We identified 88 genes that encode a total of 79 different CRPs (Table 1). Thus, the *D. melanogaster *proteome contains orthologs of all 79 mammalian CRPs (32 small subunit and 47 large subunit proteins). While the majority of CRPs are encoded by single genes, nine are encoded by two distinct genes. In addition, we identified another five genes predicted to encode proteins with significantly lower similarity to human CRPs, which we term 'CRP-like' genes. Two fragments of the *RpS6 *gene were also identified. (The list of 88 CRP genes presented by Cherry *et al*. [64] originated from an earlier report of our results to FlyBase (MA and SJM, FBrf0178764). These authors also list five CRP-like genes from our original report, but two of these have been eliminated and two additional CRP-like genes have been added in the current analysis.)

The deduced characteristics of *D. melanogaster *and human CRPs are compared in Additional data file 1. As might be expected, the amino acid identity between the CRPs of the two species is very high (average of 69% with a range of 27-98%, excluding the CRP-like proteins) and the predicted molecular weights and isoelectric points of the homologous proteins are very similar. However, several *D. melanogaster *proteins (RpL14, RpL22, RpL23A, RpL29, RpL34a, RpL34b, RpL35A) have significantly lower overall identity and different molecular weights owing to terminal deletions or extensions (data not shown; also see [65]). (If these seven proteins are discounted, the average identity of fly and human CRPs increases to 72% with a range of 43-98%.) Similar to humans and other species, there are very few acidic CRPs in *D. melanogaster*: only six proteins (RpSA, RpS12, RpS21, RpLP0, RpLP1 and RpLP2) have isoelectric points less than pH 7. (Note that RpS21 is an acidic protein, whereas its human counterpart is basic.) As in other eukaryotes, RpS27A and RpL40 are carboxyl extensions of ubiquitin [66-69], and, as in other animals, RpS30 is fused to a ubiquitin-like sequence. From these gross characterizations of component proteins, it appears that the fly cytoribosome differs only slightly from its human counterpart and is essentially the same as other eukaryotic cytoribosomes.

Previous biochemical analyses estimated that the *D. melanogaster *cytoribosome contains up to 78 CRPs [29]. This figure compares very well to the 79 different CRPs predicted by our orthology analysis (Table 1). Unfortunately, very few of the CRPs identified in the 1970s and 1980s were characterized to the level of amino acid sequence, so their correspondences to CRP genes are generally unknown, though there are a few exceptions (see references [70-73]). We have been unable, therefore, to correlate the CRPs identified in these earlier studies with those encoded by the CRP genes identified in this study. In contrast, our CRP inventory certainly does contain all 52 CRPs identified by the recent biochemical analysis of *D. melanogaster *cytoribosomes by Alonso and Santarén [34].

### Mitochondrial ribosomal protein genes

We identified 75 *D. melanogaster *genes encoding proteins of the mitoribosome (28 in the small subunit and 47 in the large subunit) by orthology to human MRPs (Table 2). These data complement and extend previous analyses of homology between human and *D. melanogaster *MRPs [16,17]. As in these previous studies, genes encoding orthologs of three human MRPs (MRPS27, MRPS36 and LACTB/MRPL56) were not found.

The MRPs of humans and *D. melanogaster *are much more divergent than are their CRPs: MRPs have an average identity of only 34% (with a range of 15-57%) and several homologous pairs differ markedly in their sizes and isoelectric points (Additional data file 2). Indeed, it is known that the mitoribosome is a rapidly evolving structure whose composition varies among eukaryotic organisms [6]. It is quite possible that there are proteins in *Drosophila *mitoribosomes that are not found in their human counterparts and these will have been missed by our orthology analysis - a definitive inventory will require biochemical characterization of the fly mitoribosome. As in mammals, three distinct genes encode three different isoforms of MRPS18 (Table 2); it is thought that each mitoribosome contains a single MRPS18 protein and that mitoribosomes may, therefore, be heterogeneous in composition [6].

### Duplicate cytoplasmic ribosomal protein genes

**Figure 1:**
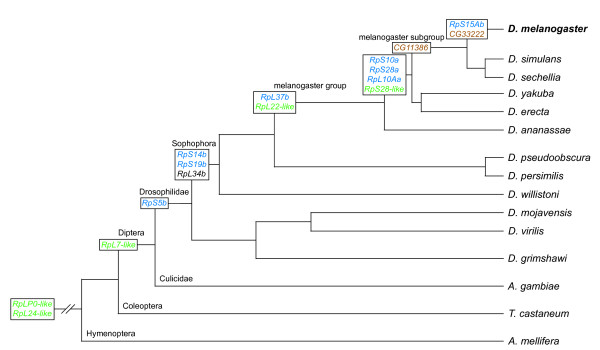
Evolution of *D. melanogaster *CRP gene duplicates and CRP-like genes. The likely pattern of emergence of CRP duplicate genes with restricted expression (blue), CRP-like genes (green) and CRP pseudogenic fragments (brown) in the lineage leading to *D. melanogaster *is shown. *RpL34b *is shown in black text: this is the only case where the newly emerged duplicate gene (*RpL34b*), rather than precursor gene (*RpL34a*), acts as the principal gene copy. The relative placement of *CG11386 *and *CG33222 *is consistent with the model presented by Stewart and Denell [86]. The dendrogram is based on that given in reference [140], in which the relationships among the Drosophilidae are taken from [149]; note that the branch lengths do not accurately reflect evolutionary time.

Of the 79 different CRPs of *D. melanogaster*, 9 are encoded by two distinct genes (Table 1). These are distinguished by a lowercase '*a*' or '*b*' suffix to the gene symbol. (The lowercase '*a*' should not be confused with the uppercase '*A*' suffix used in the standard CRP nomenclature; for example, *RpL37a *and *RpL37A *are different genes that encode different proteins.) Six of these gene pairs encode proteins of the small ribosomal subunit and the other three encode large subunit proteins. In humans, each CRP is typically encoded by a single, functional gene [37,74], but thousands of nonfunctional CRP pseudogenes are known to exist [75]. We therefore investigated the evolutionary origin, sequence conservation and expression profile of the duplicate *D. melanogaster *CRP genes in order to assess whether both members of each pair are likely to be functional (Table 3 and Figure 1).

**Table 3:** Analysis of duplicate CRP genes and CRP-like genes

	Gene			*K*_*A*_*/K*_*S*_^b^		cDNA clones		
				
	Symbol^a^	Location	Comments	Pair-wise^c^	Branch-specific^d^	Total^e^	% testis^f^	% Amino acid identity^g^
*RpS5*	*RpS5a*	X: 15E5-7	-	0.07	0.09	133	1	78
	***RpS5b***	3R: 88D6	-		0.09	60	7	
*RpS6*	*RpS6*	X: 7C2	-	NA	NA	150	5	33^h^
	***CG11386***	X: 7C2	*CG11386 *and *CG33222 *are tandem repeats of the third exon and flanking regions of *RpS6*		NA	0	0	
	***CG33222***	X: 7C2			NA	3	0	
*RpS10*	***RpS10a***	3R: 98A14	Lacks introns; likely retrogene	0.10	0.15	1	0	73
	*RpS10b*	X: 18D3	-		0.01	156	0	
*RpS14*	*RpS14a*	X: 7C6-7	*RpS14a *and *RpS14b *share identical gene structures	NA	NA	154	2	100
	***RpS14b***	X: 7C8			NA	12	0	
*RpS15A*	*RpS15Aa*	X: 11E11-12	-	0.16	0.00	150	1	98
	***RpS15Ab***	2R: 47C1	Lacks introns; likely retrogene		NA	67	1	
*RpS19*	*RpS19a*	X: 14F4	-	0.09	0.01	134	1	65
	***RpS19b***	3R: 95C13	-		0.08	1	100	
*RpS28*	***RpS28a***	3R: 99D2	Lacks introns; likely retrogene	0.05	0.06	0	0	82
	*RpS28b*	X: 8E7	-		0.00	131	1	
	***RpS28-like***	2L: 30B3	-	NA^i^	NA	1	0	36/37^j^

*RpLP0*	*RpLP0*	3L: 79B2	-	NA	NA	157	2	18
	***RpLP0-like***	2R: 46E5-6	Present in all eukaryotes		NA	11	0	
*RpL7*	*RpL7*	2L: 31B1	-	NA	NA	168	4	30
	***RpL7-like***	2L: 33C1	-		NA	32	0	
*RpL10A*	***RpL10Aa***	3R: 88D10	Lacks introns; likely retrogene	0.05	0.06	7	71	64
	*RpL10Ab*	3L: 68E1	-		0.02	160	4	
*RpL22*	*RpL22*	X: 1C4	-	NA	NA	145	2	38
	***RpL22-like***	2R: 59D3	-		NA	5	60	
*RpL24*	*RpL24*	2L: 34B10	-	NA	NA	162	3	23
	***RpL24-like***	3R: 86E5	Present in all eukaryotes		NA	34	3	
*RpL34*	***RpL34a***	3R: 96F10	*RpL34a *and *RpL34b *share identical gene structures	0.04	0.09	56	4	78
	*RpL34b*	3R: 85D15			0.04	132	2	
*RpL37*	*RpL37a*	X: 13B1	-	0.01	NA	158	3	72
	***RpL37b***	2R: 59C4	Lacks introns; likely retrogene		0.04	3	0^k^	

^a^Bold font indicates CRP-like genes, putative pseudogenic fragments (*CG11386 *and *CG33222*) or the member of a duplicate gene pair that is expressed in a small number of tissues and/or at relatively low levels. ^b^*K*_*A*_*/K*_*S *_calculations are not applicable (NA) to highly diverged sequences or cases where the numbers of both synonymous and nonsynonymous substitutions are very small (<5). ^c^Calculated for each *D. melanogaster *CRP gene pair using maximum likelihood analysis. Values for pairwise comparisons are shown on the first row of each pair. ^d^Branch-specific score in a three-way maximum likelihood tree including *D. pseudoobscura *orthologs. A four-way tree was used for *RpS19 *sequences. ^e^Total number of cDNA clones (excluding those from cultured cell lines) given in FlyBase [60] (April 2007). *RpS28-like *cDNA evidence from L Crosby (personal communication). ^f^Percentage of cDNA clones from adult testis cDNA libraries (AT, UT and BS), rounded to the nearest integer. ^g^Identity between proteins across their whole length. Values for pairwise comparisons are shown on the first row of each pair. ^h^Identity between RpS6 and the CG11386 or CG33222 protein. If *CG11386 *or *CG33222 *were used as alternative third exons of *RpS6*, the protein encoded would be 60% identical to the conventional RpS6 (see text for details). ^i^*RpS28-like *is too highly diverged from both *RpS28a *and *RpS28b *for a pair-wise *K*_*A*_*/K*_*S *_calculation to be applicable. ^j^Identity between the RpS28-like protein and RpS28a/RpS28b. ^k^There is experimental evidence that *RpL37b *expression is enriched in adult testis [79].

In five cases, one member of the gene pair lacks introns (*RpS10a*, *RpS15Ab*, *RpS28a*, *RpL10Aa *and *RpL37b*) while the other member does not. These five intronless genes are likely to have arisen by retrotransposition; that is, generated via reverse transcription of mRNA from the precursor gene followed by insertion into a new genomic location. In contrast, the *RpS5*, *RpS19 *and *RpL34 *duplicates arose through gene transposition events as both members of each pair retain introns. The *RpL34 *duplication occurred through an intrachromosomal transposition on chromosome arm 3R, and *RpL34a *and *RpL34b *have retained almost identical gene structures. In contrast, the *RpS5 *and *RpS19 *duplications involved interchromosomal transposition events that must have been followed by extensive gene remodeling as the intron-exon structures differ within each pair. Finally, *RpS14a *and *RpS14b *probably arose via unequal exchange: these paralogs are situated adjacent to each other as a tandem duplication on the *X *chromosome, share identical intron-exon structures and encode identical proteins [76]. All nine duplicate genes appear to have arisen within the Drosophilidae, albeit at different stages in the lineage leading to *D. melanogaster *(Figure 1).

Neither member of these 9 CRP gene pairs contains a nonsense mutation in the protein-coding region (data not shown), indicating that all 18 genes are potentially functional. Moreover, the low ratio of nonsynonymous to synonymous substitutions (*K*_*A*_*/K*_*S*_) between the members of each gene pair suggests that there are selective constraints on their protein-coding regions (Table 3; a *K*_*A*_*/K*_*S *_ratio significantly lower than 0.5 indicates functional constraints on both genes). Branch-specific *K*_*A*_*/K*_*S *_values further indicate that the putatively retrotransposed genes have been under overall purifying selection since their formation. Together, these data argue that none of these duplicate genes are nonfunctional pseudogenes, which is consistent with a previous analysis [77]. Indeed, the recovery of multiple cDNA clones for the majority (15/18) of these duplicate genes supports their expression *in vivo *(Table 3).

Although none of these CRP gene duplicates appear to be pseudogenes, it is evident that one member of each pair - the one with higher similarity to its human ortholog, where this difference exists (Table 1 and Additional data file 1) - is expressed at a significantly higher level and, in some cases, in a wider array of tissues than the other. This suggests that one gene of the pair produces the majority of each CRP in most cells, while the other gene has a more restricted expression pattern and, perhaps, a specialized function (indicated by bold font in Tables 1 and 3). In eight of the nine duplication events, the 'younger' gene copy has adopted the lower expression level or more restricted expression pattern; the *RpL34 *gene pair is exceptional in this regard (Figure 1 and Table 3). The expression of *RpS5b*, *RpS19b*, *RpL10Aa *and *RpL37b *appears enriched in the adult testis, suggesting the existence of testis-specific CRPs and a testis-specific cytoribosome (Table 3). Significantly, three of these genes (*RpS5b*, *RpS19b *and *RpL37b*), together with *RpS10a*, *RpS15Ab *and *RpS28a*, are autosomal copies of *X*-linked genes. These duplication events are consistent with previous studies reporting that genes with male-biased expression are predominantly autosomal [78], and that retrotransposed genes in *D. melanogaster *have preferentially retrotransposed from the *X *chromosome onto autosomes [79]. It is possible that these autosomal duplicates enable CRP expression in male germline cells, where it is hypothesized that *X *chromosome inactivation occurs during spermatogenesis [80]. Similarly, in humans, *RPS4Y *is a *Y*-linked duplicate of the *X*-linked *RPS4 *gene [10] and *RPL10L*, *RPL36AL *and *RPL39L *are autosomal retrogene copies of *X*-linked progenitors [74]. It is worth noting that expression of *D. melanogaster RpS5b*, *RpS10a *and *RpS19b *is also enriched in the germline cells of embryonic gonads [81] and/or stem cells of adult ovaries [82]. These findings suggest a germline-specific role, rather than a testis-specific role, for these CRP gene duplicates.

To conclude, the 'principal' CRPs of *D. melanogaster *- those that are expressed at high levels in most cells - are each encoded by single genes.

### Cytoplasmic ribosomal protein-like genes

We identified five *D. melanogaster *'CRP-like' genes that encode proteins with significantly lower identity to human CRPs than those described above. These are *RpS28-like*, *RpLP0-like*, *RpL7-like*, *RpL22-like *and *RpL24-like *(shown in bold font in Tables 1 and 3). Of these, RpLP0-like and RpL24-like show the most divergence from their cognate proteins, RpLP0 and RpL24. Consistent with this, *RpLP0-like *and *RpL24-like *have ancient evolutionary origins, while *RpL7-like*, *RpL22-like *and *RpS28-like *arose more recently within the Diptera (Figure 1).

cDNA evidence indicates that all five of these CRP-like genes are expressed *in vivo*, albeit at far lower levels than their cognate genes (Table 3). The evolutionary conservation of *RpLP0-like *and *RpL24-like *suggests they have important cellular functions. Indeed, the yeast ortholog of RpL24-like is found in pre-ribosomal complexes where it is thought to function in large subunit biogenesis [83]. It remains to be seen whether the other CRP-like proteins have similar functions. Interestingly, the *RpL22 *gene is *X*-linked and expressed ubiquitously, whereas *RpL22-like *is an autosomal gene that is expressed predominantly in germline cells [81,82,84,85]. This suggests that RpL22-like may have a specialized role in the germline, and perhaps within germline-specific cytoribosomes, as proposed above for some of the CRP duplicates.

*CG11386 *and *CG33222 *are 99% identical in DNA sequence and are tandem repeats of the third exon and flanking regions of the *RpS6 *gene. They likely arose via two sequential unequal crossover events [86]; the first occurring after the evolutionary split of the melanogaster subgroup, and the second being specific to *D. melanogaster *(Figure 1). Gene prediction algorithms suggest that *CG11386 *and *CG33222 *are distinct genes encoding identical amino-terminally truncated versions of RpS6 [87]; however, such proteins would lack critical functional domains and would probably be nonfunctional. In a different scenario, *CG11386 *and/or *CG33222 *could serve as alternative third exons of the *RpS6 *gene: the proteins produced would be full-length, but would differ substantially in their carboxy-terminal two-thirds from the RpS6 generated by using the conventional third exon [86]. There is, however, no direct evidence that such alternative transcripts are made. Indeed, only three cDNA clones suggest that *CG11386 *or *CG33222 *are expressed at all (Table 3). We have tentatively classified *CG11386 *and *CG33222 *as nonfunctional pseudogenic fragments.

### Chromosomal distribution of ribosomal protein genes

**Figure 2:**
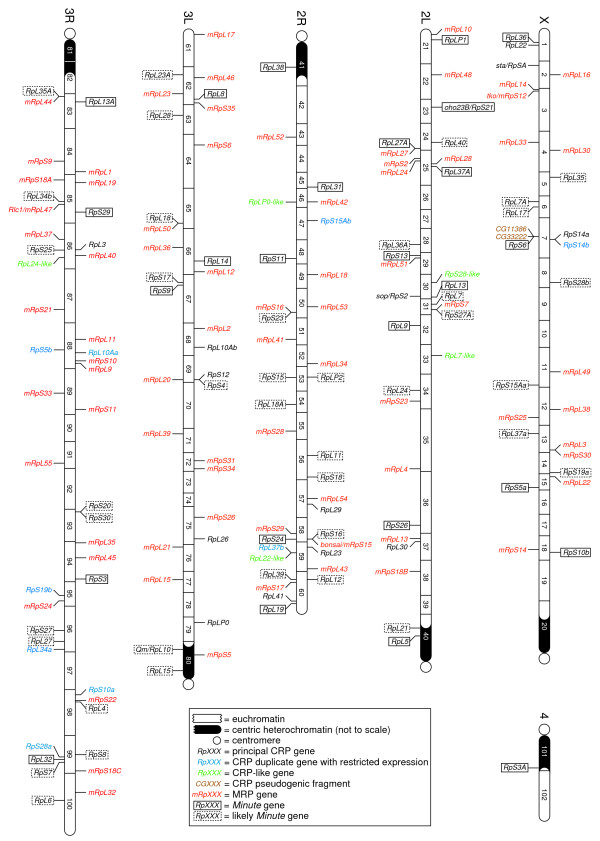
Chromosomal map of the RP genes of *D. melanogaster*. RP genes are depicted on a physical map of the genome (Release 5) [60]. Genes encoded on the positive and negative strands are shown above and below the chromosome, respectively. (The orientation of *RpL15 *is not known and its position below the chromosome is arbitrary.) Chromosomes are divided into cytological bands as determined from sequence-to-cytogenetic band correspondence tables [150]. *Minute *genes are boxed as described in the key.

As has been found for other eukaryotes [35,36,38,39], the RP genes of *D. melanogaster *are distributed across the entire genome (Figure 2). Some RP genes are tightly linked to other RP genes and, while this posed challenges for determining the phenotypes associated with individual genes (see below), we have no evidence that this distribution has functional consequences or that closely linked RP genes are transcriptionally co-regulated. Five RP genes (*RpL5*, *Qm*/*RpL10*, *RpL15*, *RpL38*, and *mRpS5*) are located within heterochromatic regions, as are certain human MRP genes [38] and some *Arabidopsis thaliana *CRP genes [36]. As heterochromatin is generally associated with the silencing of gene expression [88], the regulation of these genes must have adapted to the heterochromatic environment in order for the encoded proteins to be expressed at sufficiently high levels to meet the demand for ribosome synthesis in the cell [89].

### Ribosomal protein gene haploinsufficiency and the Minute syndrome

**Figure 3:**
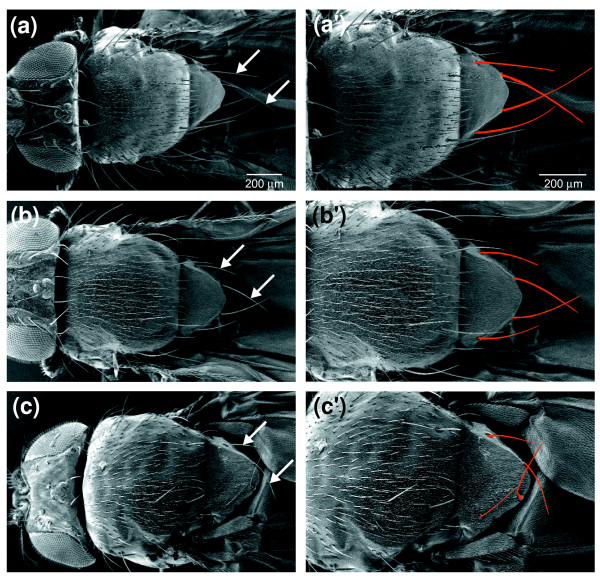
The Minute bristle phenotype. Minute flies have shorter and thinner bristles than wild type flies. This is most clearly seen by comparing the scutellar bristles, indicated here by the arrows and pseudocoloring. **(a, a') **Wild type. **(b, b') ***RpS13*^1 ^heterozygotes. **(c, c') ***RpL14*^1 ^heterozygotes.

Classical genetic studies have defined more than fifty regions of the *D. melanogaster *genome that are haploinsufficient and associated with the dominant phenotypes of prolonged development and short, thin bristles - the *Minute *loci [2] (Figure 3). To date, only fifteen *Minute *loci have been tied unequivocally to molecularly defined genes and all of these encode RPs (reviewed in reference [2]; also see references [48-53]). It has not been clear, however, if all *Minute *loci correspond to RP genes, or whether *Minute *loci may correspond to both CRP and MRP genes. We have conducted a new survey of *Minute *loci in the *D. melanogaster *genome which, combined with our RP gene inventory, has now allowed us to assess these relationships systematically.

Recent large-scale projects have provided a wealth of new genetic reagents that enable the mapping of *Minute *loci with a precision unavailable only a few years ago. Hundreds of new deletions with molecularly defined breakpoints have been provided by the efforts of the DrosDel consortium [90,91], Exelixis, Inc. [92], and the Bloomington *Drosophila *Stock Center [92]. When combined with older deletions characterized primarily through polytene chromosome cytology, these deletions have increased euchromatic genome coverage to 96-97%. In addition, transposable element insertions now exist within 0.5 kb of 57% of all genes (R Levis, personal communication), largely through the efforts of the *Drosophila *Gene Disruption Project [93] and Exelixis, Inc. [94]. We used these resources to conduct a genome-wide search for *Minute *loci. In so doing, we considered the characteristic Minute bristle phenotype (Figure 3) to be diagnostic of the Minute syndrome; we did not methodically evaluate more subtle Minute traits, such as slower development, or traits observed in only a subset of *Minute *mutants, such as impaired fecundity, reduced viability or altered body size. By combining our observations with information gleaned from published studies, we have identified 61 distinct *Minute *loci. Many of these correlate with *Minute *loci described previously (Additional data file 3), though our work has often refined their map positions. Significantly, six *Minute *loci (*M(2)31E*, *M(2)34BC*, *M(2)45F*, *M(2)50E*, *M(3)93A *and *M(3)98B*) are reported here for the first time. We also found four instances (*M(2)31A*, *M(2)53*, *M(2)58F *and *M(3)67C*) where a single *Minute *locus characterized by previous aneuploidy analyses actually comprises two separable, closely linked *Minute *genes. As we have inferred the existence of four additional *Minute *loci from patterns of deletion coverage (described below), we conclude that there are 65 distinct *Minute *loci in the *D. melanogaster *genome.

We were able to demonstrate definitively that a particular *Minute *locus corresponds to a specific RP gene when a Minute bristle phenotype was observed in one or more of the following situations: flies heterozygous for a molecularly characterized mutation in a RP gene (for example, *M(2)36F*/*RpS26*); flies heterozygous for a chromosomal deletion when the Minute phenotype could be mapped unambiguously to a single RP gene with deletion breakpoints (for example, *M(2)25C*/*RpL37A*); or flies heterozygous for a chromosomal deletion when the Minute phenotype could be rescued by a specific RP transgene (for example, *M(3)99D*/*RpL32*). We found that there are 26 unequivocally *Minute *CRP genes by these criteria (Additional data file 4; summarized in Table 4). In contrast, no MRP or CRP-like genes were definitively demonstrated to be *Minute *genes.

**Table 4:** CRP gene haploinsufficiency

CRP gene^a^		Genetic analysis^b^						
					
Symbol	Location	CRP gene mutations^c, d^	Deletions removing a single CRP gene^c, e^	Other evidence	Is the CRP gene a *Minute*?^f^	No. of candidate *Minute *genes^g^	*Minute *synonym^h^	Reference^i^
**X chromosome**								
*RpL36*	1B12		M		Yes		*M(1)1B*	[53]
*RpL22*	1C4	+	+		No			
*sta/RpSA*	2B1	+	+		No			
*RpL35*	5A11			Lies in a gap in deletion coverage. 5A6-13 aneuploids were Minute (Merriam *et al*. [55])	Likely	33	*M(1)5A*	
*RpL7A*	6B1		M		Likely	2	*M(1)5D6A*	
*RpL17*	6C10			Lies in a gap in deletion coverage	Likely	12	New?	
*RpS6*	7C2	M	M		Yes		*M(1)7BC *and *M(1)7C*	[2]
*RpS14a*	7C6-7			A deletion that removes *RpS14a *and *RpS14b *is not Minute [95]	No			
***RpS14b***	7C8			A deletion that removes *RpS14a *and *RpS14b *is not Minute [95]	No			
*RpS28b*	8E7			Lies in a gap in deletion coverage. A *Minute *mutation was mapped to 8D8-9A2 [153]	Likely	17	*M(1)8F*	
*RpS15Aa*	11E11-12		M		Likely	8	*M(1)11F*	
*RpL37a*	13B1			Lies in a gap in deletion coverage. 12F6-13B6 aneuploids were Minute (Merriam *et al*. [55])	Likely	2	*M(1)13A*	
*RpS19a*	14F4		M		Likely	14	*M(1)14E*	
*RpS5a*	15E5-7	M		Lies in a gap in deletion coverage	Yes		*M(1)15D*	[44]
*RpS10b*	18D3	M	M		Yes		*M(1)18C*	
**Chromosome arm 2L**								
*RpLP1*	21C2	M	M		Yes		*M(2)21C*	[50]
*oho23B/RpS21*	23B6	M	M		Yes		*M(2)23B*	[49]
*RpL40*	24E1		M		Likely	4	*M(2)24D*	
*RpL27A*	24F3	M	M		Yes		*M(2)24F*	
*RpL37A*	25C4		M	The interval between flanking non-Minute deletions contains only *RpL37A*	Yes		*M(2)25C*	
*RpL36A*	28D3		M		Likely	5	M(2)28DE*	
*RpS13*	29B2	M	M		Yes		M(2)29B*	[45]
*sop/RpS2*	30E1	+	+		No			
*RpL13*	30F3		M	The interval between flanking non-Minute deletions contains only *RpL13*	Yes		*M(2)31A*	
*RpL7*	31B1		M		Likely	2	*M(2)31A*	
*RpS27A*	31E1		M		Likely	6	M(2)31E*	
*RpL9*	32C1	M	M		Yes		*M(2)32D*	[46]
*RpL24*	34B10		M		Likely	8	M(2)34BC*	
*RpS26*	36F4	M	M		Yes		*M(2)36F*	
*RpL30*	37B9	+	+		No			
*RpL21*	40A-B		M		Likely	10	*M(2)39F*	
*RpL5*	40B	M	M		Yes		M(2)40B*	[51]
**Chromosome arm 2R**								
*RpL38*	41C-E	M	M		Yes		*M(2)41A*	[51]
*RpL31*	45F5	M		Lies in a gap in deletion coverage	Yes		M(2)45F*	
***RpS15Ab***	47C1		+		No			
*RpS11*	48E8-9	M		Lies in a gap in deletion coverage	Yes		M(2)48E*	
*RpS23*	50E4		M		Likely	2	M(2)50E*	
*RpS15*	53C8			*M(2)53*^1 ^Minute phenotype rescued by duplication of 51F-54A [154] but not by a *RpLP2 *transgene [155]	Likely	16	*M(2)53*	
*RpLP2*	53C9		M		Likely	5	*M(2)53*	
*RpL18A*	54C3			Lies in a gap in deletion coverage	Likely	16	New?	
*RpL11*	56D7			Lies in a gap in deletion coverage. 56C-D aneuploids were Minute [55]	Likely	3	*M(2)56CD*	
*RpS18*	56F11		M		Likely	2	*M(2)56F*	
*RpL29*	57D8		+		No			
*RpS16*	58F1			Deletions that remove both *RpS16 *and *RpS24 *are Minute. The *Minute *mutations *M(2)58F*^1 ^and *RpS24*^SH2053 ^complement, suggesting *RpS16 *is a *Minute *gene	Likely	25	*M(2)58F*	
*RpS24*	58F3	M		Deletions that remove both *RpS16 *and *RpS24 *are Minute	Yes		*M(2)58F*	
*RpL23*	59B3		+		No			
***RpL37b***	59C4		+		No			
*RpL12*	60B7			*RpL12 *and *RpL39 *lie in the same gap in deletion coverage. 60B3-10 aneuploids were Minute [156]	Likely	9	*M(2)60B*	
*RpL39*	60B7			*RpL12 *and *RpL39 *lie in the same gap in deletion coverage. 60B3-10 aneuploids were Minute [156]	Likely	9	*M(2)60B*	
*RpL41*	60E5	+	+		No			
*RpL19*	60E11	M		A deletion that removes *RpL19 *and *RpL41 *is Minute	Yes		*M(2)60E*	[42]
**Chromosome arm 3L**								
*RpL23A*	62A10		M		Likely	8	*M(3)62A*	
*RpL8*	62E7			Lies in a gap in deletion coverage containing only *RpL8*. 62E-63A aneuploids were Minute [54]	Yes		*M(3)62F*	
*RpL28*	63B14		M		Likely	10	*M(3)63B*	
*RpL18*	65E9		M		Likely	8	*M(3)65F*	
*RpL14*	66D8	M		Lies in a gap in deletion coverage	Yes		*M(3)66D*	[47]
*RpS17*	67B5			A deletion removing both *RpS17 *and *RpS9 *is Minute. The unsequenced *Minute *mutations *RpS17*^4 ^and *RpS17*^6 ^complement the *Minute *mutation *RpS9*^EP3299^, suggesting *RpS17 *is a *Minute *gene	Likely	13	*M(3)67C*	
*RpS9*	67B11	M		A deletion removing both *RpS17 *and *RpS9 *is Minute	Yes		*M(3)67C*	
*RpL10Ab*	68E1		+		No			
*RpS12*	69F5		+	A deletion that removes *RpS12 *and *RpS4 *is Minute	No			
*RpS4*	69F6			A deletion that removes *RpS12 *and *RpS4 *is Minute	Likely	2	*M(3)69E*	
*RpL26*	75E4		+		No			
*RpLP0*	79B2	+	+		No			
*Qm/RpL10*	80A		M		Likely	23	*M(3)80*	
*RpL15*	80F		M		Likely	= 11	M(3)80F*	
**Chromosome arm 3R**								
*RpL35A*	83A4			Lies in a gap in deletion coverage	Likely	3	New?	
*RpL13A*	83B6-7	M		Lies in a gap in deletion coverage	Yes		M(3)83B*	[52]
*RpL34b*	85D15			Lies in a gap in deletion coverage	Likely	3	New?	
*RpS29*	85E8	M	M		Yes		*M(3)85E*	
*RpS25*	86D8			A deletion that removes *RpS25 *and *RpL3 *is Minute	Likely	14	*M(3)86D*	
*RpL3*	86D8	+			No			
***RpS5b***	88D6	+	+		No			
***RpL10Aa***	88D10		+		No			
*RpS20*	93A1			A deletion that removes *RpS20 *and *RpS30 *is Minute	Likely	21	M(3)93A*	
*RpS30*	93A2			A deletion that removes *RpS20 *and *RpS30 *is Minute	Likely	21	M(3)93A*	
*RpS3*	94E13	M	M		Yes		*M(3)95A*	[43]
***RpS19b***	95C13		+		No			
*RpS27*	96C8		M		Likely	5	*M(3)96C*	
*RpL27*	96E9-10		M		Likely	6	*M(3)96CF*	
***RpL34a***	96F10		+		No			
***RpS10a***	98A14		+		No			
*RpL4*	98B6		M		Likely	6	M(3)98B*	
*RpS8*	99C4			Lies in a gap in deletion coverage. 99B aneuploids were Minute [157]	Likely	11	*M(3)99B*	
***RpS28a***	99D2			The Minute phenotype of a deletion removing *RpS28a *and *RpL32 *is rescued by a *RpL32 *transgene [41]	No			
*RpL32*	99D3			The Minute phenotype of a deletion removing *RpS28a *and *RpL32 *is rescued by a *RpL32 *transgene [41]	Yes		*M(3)99D*	[41]
*RpS7*	99E2			Lies in a gap in deletion coverage. 99E-F aneuploids were Minute [157]	Likely	11	*M(3)99E*	
*RpL6*	100C7			Lies in a gap in deletion coverage. 100C-F aneuploids were Minute [157]	Likely	16	*M(3)100CF*	
**Chromosome 4**								
*RpS3A*	101F1	M	M		Yes		*M(4)101*	[48]

^a^Bold font indicates the member of a duplicate gene pair that is expressed in a small number of tissues and/or at relatively low levels. ^b^Complete details are given in Additional data file 4. ^c^'M' indicates that mutation or deletion heterozygotes display a Minute bristle phenotype; '+' indicates they are wild type; a blank indicates the absence of appropriate mutations or deletions. ^d^Mutations mapped molecularly to a single CRP gene. ^e^Deletions removing several genes, but only a single CRP gene. ^f^Judged according to evidence summarized in previous three columns and presented in detail in Additional data file 4. ^g^The maximum number of genes that could correspond to the *Minute*; defined as the number of genes between the relevant deletion breakpoints minus the number of genes with non-*Minute *mutations. ^h^*Minute *synonyms from literature sources (see Additional data files 3 and 4). Asterisks indicate new synonyms assigned in this study. ^i^Reference demonstrating definite correspondence between a *Minute *locus and CRP gene. Where no reference is given, the correspondence is shown for the first time in this study.

These 26 cases of proven CRP gene-*Minute *locus correspondences provide a strong precedent for expecting that other CRP genes are also *Minute *genes. Although existing reagents do not allow us to demonstrate the correspondences definitively, we judged that a CRP gene very likely corresponds to a genetically defined *Minute *locus when one or more of the following criteria are fulfilled: a Minute phenotype is seen for a heterozygous multi-gene deletion that uncovers a single CRP gene (for example, *M(3)63B*/*RpL28*); a CRP gene lies in a gap in deletion coverage and a molecularly uncharacterized *Minute *mutation maps to the same region (for example, *M(1)8F*/*RpS28b*); or a CRP gene lies in a gap in deletion coverage and previous studies of transient aneuploids document the presence of a *Minute *locus in the same region (for example, *M(3)99E*/*RpS7*). In this way, we identified an additional 36 CRP genes that likely correspond to 34 genetically defined *Minute *loci (Additional data file 4; summarized in Table 4). Closely linked pairs of CRP genes map to the same regions as *M(2)60B *and *M(3)93A *and, as it was impossible to determine whether one or both genes of each pair are haploinsufficient, we have classified all four CRP genes as likely *Minute *genes. No CRP-like genes mapped to the regions of proven *Minute *loci. Although five MRP genes map to regions containing *Minute *loci, it is unlikely that any of them are haploinsufficient: MRP genes are not associated with Minute phenotypes in any other situation, and each of these five MRP genes is closely linked to a CRP gene (Additional data file 4).

We concluded that a further four CRP genes (*RpL17*, *RpL18A*, *RpL34b *and *RpL35A*) are likely to be *Minute *genes despite no Minute phenotype having been associated with the genomic region in which they reside. In each of these cases, the CRP gene lies in a gap in deletion coverage (Table 4, Additional data file 4), suggesting that it is a *Minute *associated with strongly reduced fertility and/or viability, which prevents the establishment of stable deletion stocks (in the absence of a corresponding duplication). Supporting this view, such severe haploinsufficiency also appears to be associated with 15 other CRP genes - all these CRP genes lie in gaps in deletion coverage and they are only considered *Minute *genes here because they have point or transposon insertion (likely hypomorphic) mutations that cause Minute phenotypes, or because they lie in regions known to harbour *Minute *loci from the phenotypes of transient aneuploids (Table 4, Additional data file 4).

For all of the 40 CRP genes classified as 'likely *Minute *genes' (through correlation with genetically proven *Minute *loci or gaps in deletion coverage), we determined the maximum number of candidate genes that could possibly account for the haploinsufficiency. We used deletions to define the smallest chromosomal interval containing the *Minute *and then eliminated genes known not to be associated with a Minute phenotype from previous studies or from our own examinations of mutant fly strains. (This task benefited greatly from the recent work of the Bloomington *Drosophila *Stock Center which, in its efforts to maximize genomic deletion coverage, has systematically generated deletions flanking haploinsufficient loci.) The number of candidate genes defined in this way was always small, ranging from 2 to 33 genes with a median of 8.5 candidate genes per *Minute *locus (Table 4, Additional data file 4). These data increase our confidence in the likely correspondences between these *Minute *loci and CRP genes.

**Figure 4:**
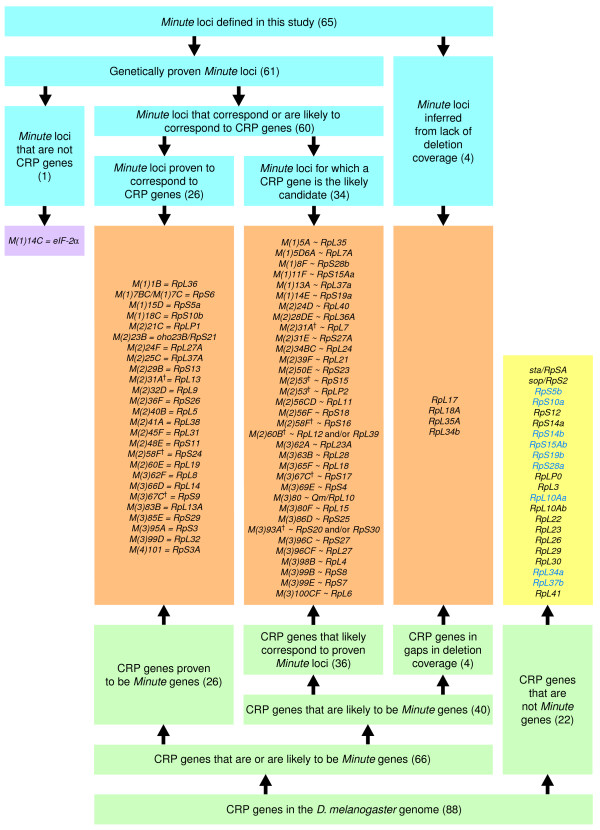
Summary of *Minute *locus - CRP gene correspondences. This figure shows the relationship between *Minute *loci defined by genetic criteria and CRP genes identified using bioinformatics. '=' indicates definite correspondence, '~' indicates probable correspondence. Daggers mark *Minute *loci that we know or strongly suspect correspond to two CRP genes (as detailed in Table 4 and Additional data file 4).

The results presented above indicate that 66 CRP genes are, or are likely to be, *Minute *genes, whereas the remaining 22 CRP genes are not (Table 4 and Additional data files 4 and 5; summarized in Figure 4). CRPs of the large and small ribosomal subunit are encoded by both *Minute *and non-*Minute *genes, with no apparent bias. Notably, none of the nine duplicate CRP genes with relatively restricted expression is a *Minute*, whereas seven of the more highly and widely expressed gene pair members are *Minute *genes. This is consistent with the idea that only one member of each of these gene pairs contributes significantly to cytoribosomal function in the majority of cells, while the one with restricted expression encodes a component of qualitatively distinct cytoribosomes in certain cell types. As the 'principal' copy of *RpS14 *or *RpL10A *is not a *Minute*, it is unsurprising that the simultaneous heterozygous deletion of both *RpS14 *genes [95] or both *RpL10A *genes (in flies with genotypes *Df(3L)ED4475*/*Df(3R)ED10556 *or *Df(3L)ED4475*/*Df(3R)ED5660*; our observations) does not produce flies with Minute phenotypes. Other possible reasons for different dosage sensitivities among CRP genes are discussed below.

The one verified *Minute *locus that does not correspond to a CRP gene is *M(1)14C*. We mapped this *Minute *gene to region 14C6 by showing that the deletions *Df(1)ED7364 *(14A8;14C6) and *Df(1)FDD-0230908 *(14C6;14E1) are each associated with a Minute phenotype. Moreover, we could rescue these phenotypes, as well as the Minute phenotype associated with the *M(1)14C*^*815-29 *^point mutation [44], using the small tandem duplication *Dp(1;1)FDDP-0024486 *(14C4;14D1). The *Minute *region defined by these experiments contains only two genes: *CG4420 *and *eIF-2α*. Significantly, flies heterozygous for *P{RS5}eIF-2α *^5-*HA*-1790^, an insertion in the 5' untranslated region (UTR) of *eIF-2α *that creates a likely hypomorphic allele, show a discernable, albeit weak Minute phenotype (our observations). This identifies *eIF-2α *as *M(1)14C*. Consistent with this conclusion, flies expressing a dominant-negative eIF-2α protein grow slowly and attain a small body size [96], phenotypes that are typical of the Minute syndrome. eIF-2α is one of the three subunits that constitute eIF2, a key translation initiation factor that delivers the methionine-loaded initiator tRNA to the ribosome by transiently associating with the small cytoribosomal subunit [97]. Although eIF-2α is not a component of cytoribosome complexes isolated by standard biochemical preparations, a reduction in *eIF-2α *gene dosage might still be expected to adversely affect cytoribosomal function and decrease overall rates of protein synthesis by specifically impairing translation initiation.

Interestingly, the gene encoding eIF-2γ, another subunit of the eIF2 translation initiation factor, is also haploinsufficient. Transcripts from the *Su(var)3-9 *gene are alternatively spliced to produce two different proteins with distinct functions: one protein is the eIF-2γ translation factor, the other is responsible for suppression of position effect variegation [98]. Mutations that specifically eliminate the suppressor protein are homozygous viable and are not associated with Minute phenotypes [99], but deletions of the entire gene are haplolethal in the absence of *P{(ry*^+^), *11. 5kb}*, a transgenic construct carrying the complete *Su(var)3-9 *genomic region (our observations; Additional data file 6). These data indicate that the regions of the *Su(var)3-9 *transcription unit encoding eIF-2γ are haplolethal. Moreover, it is possible that this haplolethality actually represents an extreme Minute phenotype associated with the *eIF-2γ*-coding regions; hypomorphic *eIF-2γ *mutants, if isolated, may show less severe Minute phenotypes.

To assess the possibility that other translation factor genes might also be haploinsufficient/*Minute*, we examined the heterozygous loss-of-function phenotypes of 68 translation factor genes we identified from BLAST searches and/or Gene Ontology classification (Additional data file 6). We identified no other cases of haploinsufficiency, though five genes could not be assessed with existing deletion and mutation strains. In contrast to the genes encoding the other two subunits of eIF2, the *eIF-2β *gene is not haploinsufficient.

As mentioned above, we have compared our inventory of *Minute *genes with the *Minute *loci defined and named from previous genetic analyses (Additional data file 3). In so doing, we failed to validate the existence of several *Minute *loci described in the past, namely *M(1)3E *[55], *M(1)4BC *[55], *M(2)21AB *[100-102], *M(2)44C *[55], *M(3)76A *[55], *M(3)82BC *[55] and *M(3)96A *[103]. The existence of some of these loci has been questioned previously and many cases appear to have involved chromosomal aberrations that were unusually complex or point mutations that were mismapped. Our failure to observe a Minute phenotype for deletions of *S-adenosylmethionine synthetase *(*Sam-S*), also known as *M(2)21AB*, is consistent with the phenotypic instability of dominant *Sam-S *mutations documented previously [100-102]. This suggests that mutations in *Sam-S *can phenocopy *Minute *mutations under certain conditions, but that *Sam-S *is not a typical *Minute *gene.

In summary, CRP genes are likely to correspond to all but one of the 65 *Minute *loci defined in this study, with the sole exception encoding a translation initiation factor subunit (Figure 4). No MRP or CRP-like genes are unequivocally associated with a Minute phenotype, indicating that the Minute syndrome is specifically related to the function of the cytoribosomes responsible for the majority of cellular protein translation, rather than the function of specialized cytoribosomal variants. Twenty-five percent of CRP genes are not associated with an obvious haploinsufficient phenotype, clearly reinforcing previous findings that not all CRP genes are *Minute *genes [61,95,104].

## Discussion

### CRP gene haploinsufficiency and the cytoribosome

When one examines the phenotypes of flies carrying chromosomal deletions, one is struck by the remarkable tolerance of *Drosophila *to aneuploidy: flies heterozygous for deletions of hundreds of kilobases of DNA usually have no obvious dominant phenotypes. For this reason, the haploinsufficiency of single genes is all the more remarkable. It is even more striking that the vast majority of these haploinsufficient genes encode proteins of the cytoribosome and that haploinsufficiency is not apparent for genes encoding components of equally elaborate cellular complexes, such as mitoribosomes or spliceosomes. What accounts for the exquisite dosage sensitivity of CRP genes?

The primary cause of CRP gene haploinsufficiency is reasonably clear: halving the copy number of a CRP gene results in reduced mRNA expression of that CRP [105]. Similarly, depleting CRP mRNAs through antisense- or RNA interference (RNAi)-mediated approaches can also produce Minute phenotypes [106,107] (SJM and SJL, unpublished data). As there appear to be no compensatory increases in transcription [105], reducing dosages of CRP genes must result in reduced CRP protein levels in the absence of dramatic changes in mRNA stability or CRP protein turnover. How then does the reduction in the level of a single CRP result in impaired cytoribosomal function and reduced general protein synthesis, and how is this manifested as the Minute phenotype?

One possibility, termed the 'balance hypothesis' [108,109], is linked to the multisubunit nature of the cytoribosome. It posits that an imbalance in the concentrations of CRPs results in the assembly of incomplete and non-functional ribosomal subunits. Indeed, it is known that depletion of individual CRPs in *Saccharomyces cerevisiae *causes inefficient ribosomal subunit assembly and/or function [110,111]. Nevertheless, the balance hypothesis also predicts that overexpression of individual CRPs should cause stoichiometric imbalances and phenotypes similar to those produced by underexpression. This prediction is not upheld in either *S. cerevisiae *[112] or *D. melanogaster *[113]. Consequently, imbalance *per se *cannot account for the haploinsufficiency of CRP genes.

A simpler explanation of CRP haploinsufficiency is that a high concentration of cytoribosomes is required for proper cellular functions and that the cytoribosome population decreases sharply when the level of a single CRP is reduced. Cytoribosomes and their components do appear to be required in unusually high quantities: CRP mRNAs are among the most abundant cellular transcripts both in yeast [114] and in flies [60], and can account for 50% of all RNA polymerase II-mediated transcription [89]. What seems critical, however, to the high-level production of fully formed cytoribosomes is that the concentration of each and every CRP never falls below a minimal level. In other words, cytoribosomal assembly is strictly limited by the availability of the least abundant component. This is probably not just a matter of simple self-assembly kinetics as improperly assembled ribosomal subunits and excess CRPs are actively degraded in yeast [111,115]. Similarly, RNAi-mediated depletion of single CRPs leads to the depletion of other CRPs in flies [64], suggesting the existence of similar degradation processes. Consequently, halving the supply of a single, limiting CRP is expected to halve the number of functional cytoribosomes. This may be tolerated by many cellular processes but will have severe effects wherever high protein synthesis is required, such as bristle formation and oocyte production in flies, or growth of *S. cerevisiae *in rich media [116]. It appears, therefore, that it is the combination of high demand for cytoribosomes and an assembly mechanism that assures that the level of the least abundant CRP determines the final concentration of cytoribosomes that makes CRP genes so exquisitely and specifically dosage sensitive. This perspective also provides a context for understanding the non-additivity of *Minute *mutations [117], where combinations of *Minute *mutations usually do not have a cumulative effect, but rather result in a phenotype similar to that of the most severe individual *Minute *mutant.

If adequate levels of cytoribosomes depend not so much on precise equimolar CRP concentrations as a minimal concentration of each and every CRP, then we should expect that variation in the expression of different CRPs (above the minimum level) might normally be tolerated *in vivo*. Such variation could be the result of differences in rates of gene transcription, mRNA translation, or mRNA/protein stability. This view provides a framework for understanding the spectrum of haploinsufficient phenotypes associated with CRP genes, which ranges from no obvious phenotypes, through bristle defects and reduced fecundity and viability, to dominant sterility or haplolethality in the most severe cases. That is, the severity of Minute phenotypes may be related to the rates at which individual CRPs are normally produced [105,106].

In reality, the explanation of CRP gene haploinsufficiency is probably more complicated than cytoribosome assembly relying simply on minimal CRP concentrations. The exact function, position or stoichiometry of CRPs within the cytoribosome may determine whether its gene is haploinsufficient and the severity of the Minute phenotype. For instance, our finding that the gene encoding the eIF-2α translation initiation factor subunit is a *Minute *could indicate that haploinsufficient CRP genes encode ribosomal components involved specifically in translation initiation. As another example, RpLP1 and RpLP2 are the only CRPs required in two copies per cytoribosome [15] and, consequently, they must be produced at twice the level of all other CRPs. It is perhaps not surprising, therefore, that both *RpLP1 *and *RpLP2 *are haploinsufficient (Table 4) [50]. It may even be the case that the haploinsufficiency of some CRP genes arises by less conventional mechanisms. For example, the introns of 27 CRP genes host genes for small nucleolar RNAs (snoRNAs) [118-122], a class of non-coding RNAs that guide post-transcriptional modifications of rRNA molecules necessary for the maturation and incorporation of rRNA into ribosomes [123]. The expression of intronic snoRNAs depends upon the expression and processing of mRNAs from the host gene [124]. Consequently, mutations that reduce expression or splicing of CRP transcripts harboring snoRNAs will simultaneously deplete the cell of both a CRP and properly processed rRNA molecules, thereby impairing cytoribosome biogenesis in two different ways. Although 21 of the 27 CRP genes that carry snoRNA genes within their introns are *Minute *or likely *Minute *genes (data not shown), the presence of intronic snoRNA genes cannot be the sole factor determining CRP gene haploinsufficiency. Indeed, we have not found any single factor that clearly determines the degree of dosage sensitivity exhibited by different CRP genes.

Regardless of the exact causes and mechanisms of haploinsufficiency, it is pertinent to ask why the majority of CRPs are expressed so close to the level of sufficiency, such that loss of one gene copy is debilitating, rather than being synthesized in excess? One possibility concerns economics: cytoribosomal synthesis is an incredibly costly affair [89] and excessive CRP production would both be wasteful and monopolize the limited resources of the cell. A second possibility is that CRP levels are normally constrained to guard against inappropriate activation of cell growth, proliferation or apoptosis - processes in which CRPs and cytoribosomes have been postulated to play direct roles [125]. A final and intriguing possibility is that the barely sufficient expression levels of some CRP genes may have evolved as a viral defense mechanism. Cherry *et al*. [64] found that reducing the levels of 64 of the 79 principal CRPs by RNAi inhibits the propagation of *Drosophila *C virus in *Drosophila *adults and cultured cells. Because this virus requires high concentrations of cytoribosomes in its host cell to undergo efficient translation, tightly controlled expression of CRP genes at levels just sufficient for normal growth and development may protect against viral infection and provide a selective advantage. Interestingly, we found a modest correlation between a CRP gene being a *Minute *and it being able to inhibit virus replication in this assay. Clearly, further work will be required to test whether there is truly a relationship between normal CRP gene expression levels and susceptibility to viruses.

*Minute *mutations attracted the attention of early geneticists because they were isolated so often in *D. melanogaster*. In fact, Schultz said in 1929, "...so many have been found that this mutant type is one of the most frequent in *Drosophila*" [117]. As a considerable number of *Minute *mutants have also been isolated in other *Drosophila *species [60], one might be justified in thinking *Drosophila *are unusually sensitive to CRP gene haploinsufficiency. On the other hand, the phenotypic consequences of CRP gene haploinsufficiency may simply be more noticeable in flies because they include conspicuous changes in external morphology. In fact, 'Minute-ness' may be a widespread phenomenon that is under-recognized because CRP gene haploinsufficiency has different and varied phenotypic consequences in other organisms. Recent research suggests this is the case [116,126-132]. For example, *RPS5 *haploinsufficiency disrupts cell division and causes developmental and growth phenotypes in *Arabidopsis *[126]; several CRP genes are haploinsufficient for suppression of nerve sheath tumors in zebrafish [127]; and *RPS19 *haploinsufficiency is a causative factor of Diamond-Blackfan anemia in humans [128,130]. In fact, our reliance on the obvious bristle phenotype to distinguish *Minute *from non-*Minute *loci may present a biased assessment of CRP gene haploinsufficiency in the fly: it is quite possible that the 22 CRP genes classified as non-*Minute *in this study are associated with more subtle haploinsufficient phenotypes. How reduced CRP expression gives rise to diverse phenotypes is a mystery that, at least in part, reflects our current ignorance of the regulation and roles of CRPs in different cell types. This is certainly a topic worthy of more research.

## Conclusion

We have assessed an idea that has been discussed for more than three decades; namely, that the haploinsufficient *Minute *loci of *Drosophila *correspond to the genes encoding protein components of ribosomes [2,133]. Our results confirm this idea and add important details. We have shown that *Minute *genes encode proteins of cytoplasmic ribosomes and not mitochondrial ribosomes, and we have defined the subset of CRP genes that are haploinsufficient. While duplicate genes encoding tissue-specific CRPs are not associated with Minute phenotypes, it is not otherwise clear what distinguishes the CRP genes that are haploinsufficient from those that are not. We identified a single *Minute *gene encoding a different kind of protein, a cytoplasmic translation initiation factor subunit. This hints that haploinsufficient CRP genes may encode proteins specifically involved in translation initiation, although further work is obviously needed to test this idea.

*Minute *genes account for the vast majority of the haploinsufficient genes in the *D. melanogaster *genome with effects on fertility and viability strong enough to prevent the recovery of chromosomal deletions in the absence of corresponding duplications. Indeed, there are very few additional genes (for example, *dpp *[134], *Abd-B *[135]) or chromosomal regions (for example, *Tpl *[136], *wupA *[137], *Fs(1)10A *[138]) unequivocally associated with haplolethality or haplosterility. (A few other regions have been reported but not investigated in detail.) The most immediate practical use for our data will be in systematic efforts to maximize genome deletion coverage. Knowing which specific genes are haploinsufficient will make it feasible to flank each one as closely as possible with pairs of deletions, or to delete these genes in the presence of duplications or transgenic rescue constructs. Further improvements in deletion coverage will undoubtedly identify and map the remaining haplolethal or haplosterile loci.

Collectively, our inventories of the RP genes and *Minute *loci of *D. melanogaster *provide a solid foundation for further studies of RPs, ribosomes, and the causes and consequences of haploinsufficiency in flies and other organisms.

## Materials and methods

### Bioinformatics

RefSeq human RP sequences were obtained from the National Center for Biotechnology Information [139]. The FlyBase BLASTp service [140] was used to identify high scoring hits from among the annotated proteins of *D. melanogaster*; tBLASTn was used when orthologs were not identified by a BLASTp search. The ExPASy proteomics server [141] was used to compute the average pI and molecular weight of the RPs. The percentage identity between human and *D. melanogaster *RP sequences or between *D. melanogaster *RP pairs was calculated using the NPS@ ClustalW alignment tool at the Pôle Bioinformatique Lyonnais using default parameters [142,143]. *K*_*A*_/*K*_*S *_values were estimated using the program package PAML [144]. cDNA clone data were obtained from FlyBase [60].

The identification of CRP gene orthologs and the plotting of their evolutionary emergence (Figure 1) were achieved using a combinatorial approach. First, sequences corresponding to the relevant CRP genes from *D. melanogaster *and *Homo sapiens *were used as queries in BLASTn, BLASTp, tBLASTn and BLAT searches of the genomes of other Drosophilid and insect species using the FlyBase BLAST server [140] and the UCSC Genome Bioinformatics BLAT server [145,146]. High scoring matches were judged to be potential orthologs and were analyzed further using the FlyBase OrthoView tool [87]. Second, the coding sequences (CDS) of relevant CRP genes from *D. melanogaster *and *H. sapiens *were used as queries in BLASTn searches of the GLEANR CDS prediction sets of other Drosophilid species [140]; phylogenetic trees were then generated from the high scoring matches, with the CDS of *S. cerevisisae *CRPs as roots, using the ClustalW tools at EMBL-EBI [147]. Third, NCBI Homologene [148] was searched for any relevant homology calls: *RpLP0-like*, *RpL7-like *and *RpL24-like *were found in HomoloGene clusters 102093, 64526 and 9462, respectively. The results of all these analyses were then compared, with the most parsimonious interpretations being used to annotate the dendrogram shown in Figure 1.

### Assessing Minute phenotypes and mapping *Minute *mutations

In order to compile a list of all genetically defined *Minute *loci, we first catalogued all the *Minute *loci described in the fly literature [2,54,55,60]. We then inspected deletions for all genomic regions having deletion coverage to confirm or refute the existence of these *Minute *loci and to identify any new *Minute *loci that had previously gone undetected. Minute phenotypes were scored primarily by visual inspection of bristle length, although body size, developmental timing, fertility and viability were considered when information was available. Deletion-bearing flies were outcrossed to Oregon-R or Canton-S wild-type strains whenever we could not unambiguously score Minute phenotypes in stocks. The phenotypic effects of deleting or disrupting *X*-linked genes were assessed only in heterozygous females. Finally, the cytological locations of all verified *Minute *loci were correlated with the positions of RP genes to identify candidate genes.

To assess RP gene haploinsufficiency directly, we inspected flies heterozygous for deletions and/or mutations of molecularly identified RP genes. Minute phenotypes were scored as described above. For some deletions that had not been characterized molecularly, it was necessary to refine the mapping of breakpoints with complementation tests against molecularly mapped mutations or with polytene chromosome preparations to determine whether RP genes were deleted. (In a few cases, RP genes were classified as lacking deletion coverage when the only existing deletions were not useful in a practical sense owing to their associated chromosomal rearrangements or extremely large size.) A transposable element insertion was judged to disrupt a RP gene if it failed to complement other mutations in the gene, if we saw a Minute phenotype in the insertion strain, or if the transposon is inserted in the protein-coding region or 5' UTR of the gene based on FlyBase annotations [87] or our own BLAST analyses. (By these criteria, many nearby insertions, intronic insertions, and insertions in 3' UTRs were not used in our analysis.) We included molecularly characterized point mutations in our analyses for the few RP genes where they were available. Molecularly uncharacterized *Minute *point mutations from the Bloomington Stock Center were complementation tested against mutations and deletions known to disrupt or delete specific RP genes.

Fly strains were obtained from the Bloomington, Szeged, Kyoto and Harvard *Drosophila *Stock Centers. Helene Doerflinger and Daniel St Johnston provided *Df(3R)IR16 *and *Df(3R)MR22 *stocks, and Yuri Sedkov and Alexander Mazo provided *mRpL16*^*A*^, *mRpL16*^*B *^and *mRpL16*^*C *^stocks.

## Abbreviations

CDS, coding sequence; CRP, cytoplasmic ribosomal protein; MRP, mitochondrial ribosomal protein; RNAi, RNA interference; RP, ribosomal protein; rRNA, ribosomal RNA; snoRNA, small nucleolar RNA; UTR, untranslated region.

## Authors' contributions

SJM, JR, GR, AL and KRC conceived, designed and performed the experiments. SJM, MA, PH, ZY and KRC analyzed the data. JR, GR and KRC contributed fly strains. MA, GM and NK helped with gene classification and nomenclature. TCK and SJL provided funds, lab space and general support. SJM and KRC wrote the paper.

## Additional data files

The following additional data are available with the online version of this paper. Additional data file 1 is a table comparing the physical characteristics of *D. melanogaster *and human CRPs, together with their RefSeq accession numbers. Additional data file 2 is a similar table comparing *D. melanogaster *and human MRPs. Additional data file 3 is a table listing all the *Minute *loci in a historical context. Additional data file 4 is a table showing our comprehensive genetic analyses of ribosomal protein gene haploinsufficiency. Additional data file 5 is a table listing CRP gene-*Minute *locus correspondences arranged in alpha-numerical order by RP gene symbol. Additional data file 6 is a table showing our genetic analyses of translation factor gene haploinsufficiency.

## Supplementary Material

Additional File 1 Comparison of the physical characteristics of *D. melanogaster *and human CRPs, together with their RefSeq accession numbers. Click here for file

Additional File 2 Comparison of the physical characteristics of *D. melanogaster *and human MRPs, together with their RefSeq accession numbers. Click here for file

Additional File 3 Listing of all the *Minute *loci in a historical context. Click here for file

Additional File 4 Comprehensive genetic analyses of ribosomal protein gene haploinsufficiency. Click here for file

Additional File 5 CRP gene-*Minute *locus correspondences arranged in alpha-numerical order by RP gene symbol. Click here for file

Additional File 6 Genetic analyses of translation factor gene haploinsufficiency. Click here for file
